# Numerical Analysis of the Transport and Fate of Nitrate in the Soil and Nitrate Leaching to Drains

**DOI:** 10.1100/tsw.2001.344

**Published:** 2001-12-01

**Authors:** Alaa El-Sadek, Mona Radwan, Jan Feyen

**Affiliations:** Katholieke Universiteit Leuven, Hyddrauliccs Laboratory, Faculty of Engineering, Heverlee, Belgium

**Keywords:** DRAINMOD, nitrate transport, nitrate leaching, environment

## Abstract

In this study, the transport and fate of nitrate within the soil profile and nitrate leaching to drains were analyzed by comparing historic field data with the simulation results of the DRAINMOD model. The nitrogen version of DRAINMOD was used to simulate the performance of the nitrogen transport and transformation of the Hooibeekhoeve experiment, situated in the sandy region of the Kempen (Belgium) and conducted for a 30-year (1969–1998) period. In the analysis, a continuous cropping with maize was assumed. Comparisons between experimentally measured and simulated state variables indicate that the nitrate concentrations in the soil and nitrate leaching to drains are controlled by the fertilizer practice, the initial conditions, and the rainfall depth and distribution. Furthermore, the study reveals that the model used gives a fair description of the nitrogen dynamics in the soil and subsurface drainage at field scale. From the comparative analysis between experimental data and simulation results it can also be concluded that the model after calibration is a useful tool to optimize as a function of the combination “climate-crop-soil-bottom boundary condition” the nitrogen application strategy resulting in an acceptable level of nitrate leaching for the environment.

## INTRODUCTION

In western Europe, the current nitrogen use leads to considerable nitrate-nitrogen ($NO_3$–N) losses through leaching. The nitrate remaining in the soil after harvest, plus nitrate derived from mineralization of organic nitrogen in soil, organic material, plant residue, or organic manure, is at considerable risk of being lost by leaching during the subsequent winter. The nitrate directive defines a maximum admissible concentration of 11.3 mg $NO_3$–N $l^{-1}$ in surface water and groundwater. In order not to exceed this European Union limit, agriculture in Flanders is under severe pressure. At present, the fertilizer regulation in Flanders states that the residual $NO_3$–N, measured in the soil profile from 0 to 90 cm, may not exceed 90 kg $NO_3$–N $ha^{-1}$ between October 1 and November 15[1].

The various nitrogen processes occurring within the soil profile play a major role in determining the nitrate leaching to surface and groundwater. The objective of this article is to study for a long period the transport and fate of nitrate within the soil profile and nitrate leaching to drains, applying the methodology to the Hooibeekhoeve experiment, situated in the sandy region of the Kempen (Belgium) for a 30-year (1969–1998) period. Within this frame the comparison between field data and simulation results obtained with the DRAINMOD model is also given.

## METHOD

The DRAINMOD[2] model (Fig. 1) was used to simulate the Hooibeekhoeve experiment, the performance of the drainage system, and the impact of the related water-table management. DRAINMOD-N[3,4], an add-on module to DRAINMOD for simulating the nitrogen dynamics in drained soils, was used to model the $NO_3$–N leaching from the root zone into the subsurface drainage system. $NO_3$–N is the main nitrogen pool considered. The model is a quasi two-dimensional model because the nitrogen movement component considers only vertical transport in the unsaturated zone and both vertical and lateral transport in the saturated zone. The controlling processes considered by the model[5] are rainfall deposition, fertilizer dissolution, net mineralization of organic nitrogen, denitrification, plant uptake, and surface runoff and subsurface drainage losses. The main components and processes of the water and nitrogen balance, presented in the DRAINMOD/DRAINMOD-N model, are schematically presented in Figs. 1 and 2, respectively.

## MATERIALS

At the "Hooibeekhoeve" in the community of Geel (northeastern part of Belgium), an experimental field trial with maize was set up by the Belgian Soil Service[6] from 1992 to 1995. The soil at the farm site is sandy and classified as a Haplic Podzol, a moderately wet sandy soil with a distinct humus and/or iron B horizon

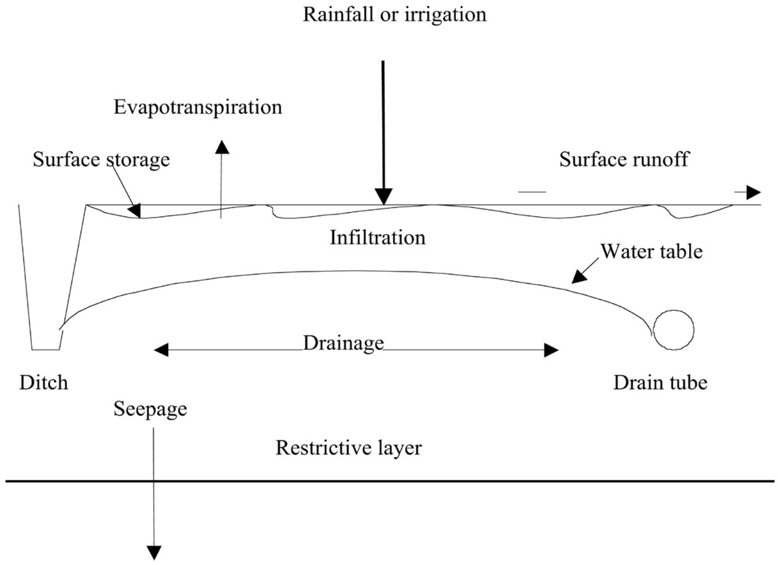

**FIGURE 1.** An overview of components in the water balance of the DRAINMOD model.

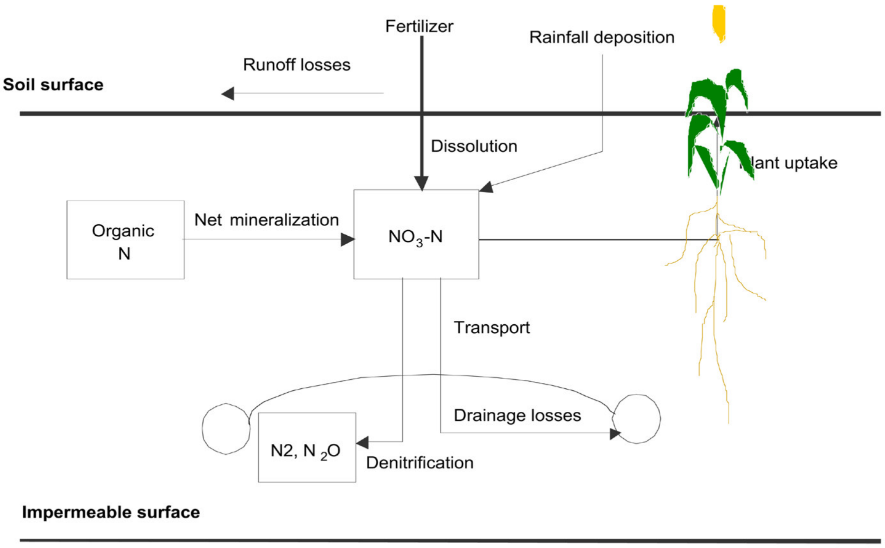

**FIGURE 2.** Schematic presentation of the nitrogen cycle as described in DRAINMOD-N.

(a Zdg soil according to the Belgian Soil Classification System). The groundwater level fluctuates between 115 and 160 cm below surface. During the first 2 years of the experiment, in 1993 and 1994, maize was cultivated, whereas in the last season, 1995, the field was left fallow. Different pig slurry fertilizer application packages were applied in spring or autumn. The fertilizer scenarios are listed in Table 1. $NO_3$–N in the fertilizer package is added to the soil solution by dissolution of the fertilizer.

The physical properties of the soil were determined in one plot of the Hooibeekhoeve for each distinguishable soil horizon, using undisturbed soil samples taken with Kopecky rings. Van Genuchten/Mualem parameters for describing the hydraulic functions[7] were fitted on both water-retention and multistep outflow data, using the multistep outflow program[8]. Basic water-retention and hydraulic conductivity curves were established by averaging individual curves for each soil layer. In addition, the soil texture was measured for each soil horizon[9]. The soil physical properties are listed in Table 2. The field was intensively monitored during the experimental period. Every 3 weeks, soil samples were taken with an interval of 30 cm to a depth of 120 cm for mineral nitrogen measurements. Mineral nitrogen was measured in groundwater at 200 cm with the same interval.

During the simulation period the field was cropped with maize. Only organic manure was applied as fertilizer. Missing data required to run the model was either supplementarily measured or reconstructed by using the pedotransfer functions of Vereecken[10], as indicated by Ducheyne and Feyen[9]. The 3-year data (1992–1995) were used to extensively calibrate and validate the DRAINMOD/DRAINMOD-N models.

In the scenario analysis that was carried out for the period from 1969 to 1998, the properties of the field plot of the Hooibeekhoeve (used for model calibration and validation) were used. Further it was assumed that the field was equipped with a subsurface drainage system consisting of parallel, 10-cm (diameter), corrugated plastic drains. The drainage design consisted of drain spacing of 25 m and drain depth of 1.25 m. The management treatment was conventional (drains with free outlet, not submerged). The total fertilizer package considered in the scenario analysis, applied in two dressings, was 150 kg N $ha^{-1}$. Detailed inputs for the maize production practices and $NO_3$–N

**TABLE 1**
**Field Treatments**

| Treatment | Description of Fertilizer Package |
|---|---|
| 1 | 30 ton $ha^{-1}$ pig slurry applied in autumn |
| 2 | 120 ton $ha^{-1}$ pig slurry applied in autumn |
| 3 | 30 ton $ha^{-1}$ pig slurry applied in spring |
| 4 | 120 ton $ha^{-1}$ pig slurry applied in spring |
| 5 | 60 ton $ha^{-1}$ pig slurry applied in autumn + 60 ton $ha^{-1}$ applied in spring |

**TABLE 2**
**Soil Physical Properties of the Experimental Field at the Hooibeekhoeve**

| | Soil Horizon (cm) | | | |
|---|---|---|---|---|
| Soil Parameter | 0–35 | 35–50 | 50–100 | 100–200 |
| van Genuchten | | | | |
| $\theta_r$ | 0.055 | 0.019 | 0.011 | 0.017 |
| $\theta_s$ | 0.48 | 0.43 | 0.42 | 0.42 |
| $\alpha$ | 0.016 | 0.028 | 0.032 | 0.019 |
| m | 1.574 | 1.686 | 1.85 | 1.804 |
| n | 0.365 | 0.407 | 0.459 | 0.446 |
| Mualem | | | | |
| $K_{sat}$, cm $day^{-1}$ | 50.47 | 18.6 | 15.55 | 13.15 |
| $\lambda$cm $day^{-1}$ | 2.031 | 4.041 | 3.314 | 4.007 |
| Soil texture (%) | | | | |
| Clay | 2 | 7 | 2 | 2 |
| Silt | 5 | 16 | 3 | 3 |
| Sand | 93 | 77 | 95 | 95 |

transport and transformation variables are listed in Table 3. The maize production practices used in the simulations are characteristic for the sandy region of the Kempen[11].

## RESULTS AND DISCUSSIONS

Water quality modeling is dependent upon the success of water quantity modeling driven by hydrology and hydraulics[12]. Planners and decision makers in water resources management therefore try to simulate as accurately as possible the water quantity and use this as starting point for the modeling of the water quality aspects. Within this context, a good water table prediction is a necessity to ensure good prediction of nitrate leaching. The comparison between the simulated and measured water table is given in Fig. 3; the goodness of fit is shown in Fig. 4. Assuming that the water is the vehicle carrying nitrate through the soil profile, results of Figs. 3 and 4 ensure a good nitrate transport and leaching as a second step.

The sensitivity analysis performed for the DRAINMOD-N model (done in a previous study[13]) indicated that maximum $NO_3$–N concentrations in the soil solution were found to be sensitive to the mineralization standard rate coefficient and nitrogen content in crop (maize), especially at low-to-medium depths (60 cm or less), and to the denitrification standard rate coefficient and dispersivity, especially at medium depths (30 to 60 cm). At larger depths in the profile maximum $NO_3$–N concentrations are insensitive to all parameters. The sensitivity analysis results, with respect to the subsurface drainage modeling, indicated that $NO_3$–N loss in subsurface drainage is most sensitive to the standard rate coefficients for denitrification and mineralization, mildly sensitive to nitrogen content in the crops, and insensitive to $NO_3$–N content in rainfall and dispersivity (these results are similar to those reported for conditions in eastern North Carolina by Brevé et al.[3]). Based on the results of the sensitivity analysis, only the standard rate coefficients for denitrification and mineralization were further optimized.

**TABLE 3**
**Summary of Inputs for DRAINMOD-N**

| | |
|---|---|
| **Soil Properties** | |
| $\theta_{wp}$ ($cm^3\ cm^{-3}$) | 0.17 |
| Bulk density ($g\ cm^{-3}$) | 1.6 |
| Organic nitrogen in top soil ($\mu g\ g^{-1}$) | 3200 |
| $K_{mnl}$ ($day^{-1}$) | $3.5 \times 10^{-5}$ |
| $K_{den}$ ($day^{-1}$) | 0.10 |
| **Drainage System Parameters** | |
| Drain depth (m) | 1.25 |
| Drain spacing (m) | 25 |
| Surface storage (cm) | 2.5 |
| Effective drain radius (cm) | 2.5 |
| **Maize Production Parameters** | |
| Desired planting date | May 4 |
| Length of growing season (day) | 120 |
| N-fertilizer input (kg N $ha^{-1}$) | 150 |
| Date fertilizer application | May 6, May 14 |
| Depth fertilizer incorporated (cm) | 10 |
| Total dry matter production (kg $ha^{-1}$) | 18,000 |
| **Other Nitrogen Model Parameters** | |
| Dispersivity (cm) | 10 |
| $NO_3$–N content of plant (%) | 1.5 |
| $NO_3$–N concentration of rain (mg $l^{-1}$) | 0.8 |

The soil, crop, and nitrogen parameters were calibrated resulting in a set of representative parameters for the given soil-crop condition. The calibration of the model parameters was done by trial and error[14]. The calibration of the DRAINMOD-N model is based on field data of the fertilizer scenario number 3 (30 ton $ha^{-1}$ pig slurry applied in spring) (Table 1). The calibrated model (DRAINMOD-N) was validated vs. data collected on the field fertilizer scenario number 5 and applied to simulate the nitrate transport in the soil profile for the other scenarios (1, 2, and 4).

The complete results of the calibration, validation, and scenario analysis are given in Figs. 5, 6, and 7, respectively. The confrontation of the simulation results to experimental data (Figs. 3, 4, 5, 6, and 7) reveals that the DRAINMOD model can acceptably simulate the water flux and the nitrate transport within the soil profile provided the model parameters and other input data are available. The validation and scenario-analysis tests showed that the model yields good estimates of the nitrate concentrations in the soil.

The statistical results presented in the Table 4 clearly illustrate that the DRAINMOD-N model performs well in the calibration and validation phase, as well as when the model is used in a predictive mode. The first column in Table 4 indicates that the statistical results of the goodness of fit and root mean square residual at 30- to 60-cm depth and bottom of the root zone (90 to 120 cm) are given for calibration (scenario 3), for validation (scenario 5), and for scenario analysis (scenarios 1, 2, and 4), respectively. It is evident that when the tonnage of manure applied increases, the amount of nitrate leaching at the bottom of the root zone increases and exceeds legal limits.

After having calibration and validation of the models, a scenario analysis was performed to assess the effect of changes in weather data on the $NO_3$–N leaching. As Brevé et al.[4] found, analysis of the simulation results showed also that the mineralization rate reduces when the water table is close to the surface and during extended dry periods. $NO_3$–N is depleted from the soil solution by denitrification, plant uptake, and drainage and runoff losses. Denitrification increases during wet periods when the water table is high but is negligible during dry periods. Although plant uptake occurs throughout the entire growing season, $NO_3$–N losses in surface runoff are bound to rainy periods. The study also implies that higher fertilizer applications result in higher nitrate concentrations patterns, with more elevated risks if fertilizer is applied in autumn.

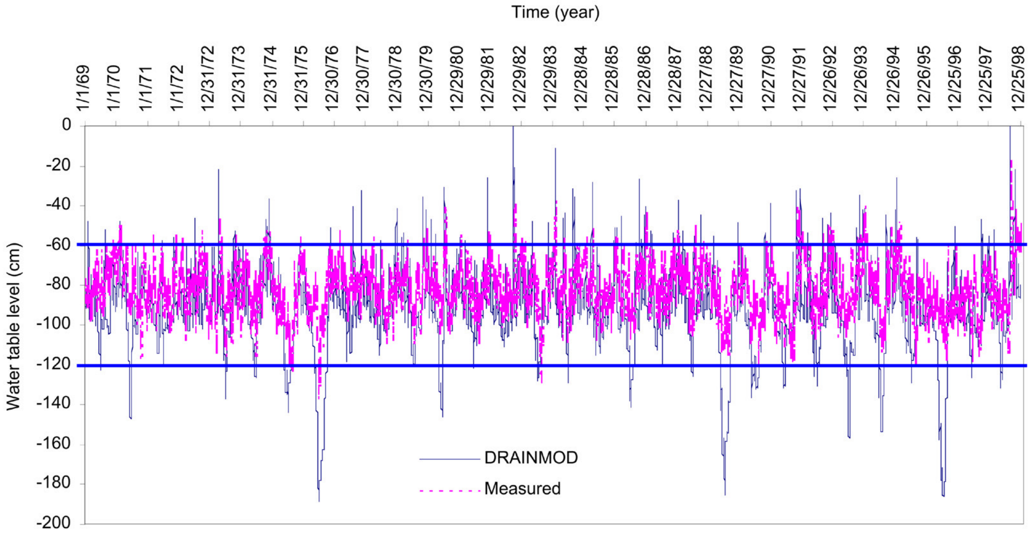

**FIGURE 3.** Simulated and measured water table level.

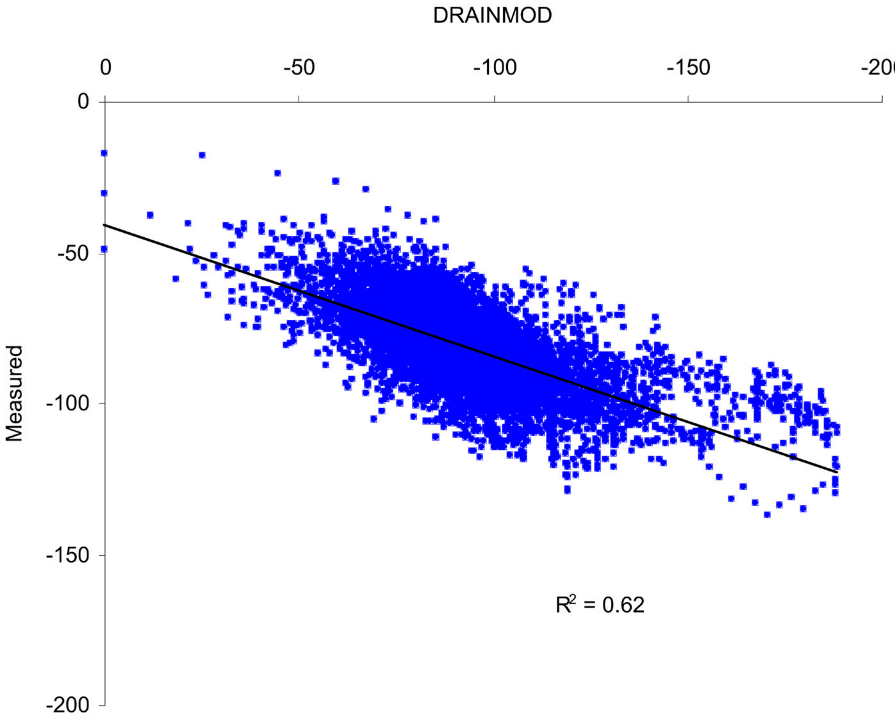

**FIGURE 4.** The goodness of fit ($R^2$) between simulated and measured water table level (cm).

Given the uncertainty of inputs and model parameters, the simulation results of the scenario analysis should be interpreted in a relative, rather than an exact, way. The simulation results indicate that increasing the mineralization amount results in an increase of both plant uptake and nitrate loss. The results also show that an increase in the denitrification process results in a reduction in the amount of $NO_3$–N leached. On the other hand, a corresponding reduction in the plant uptake is calculated.

The simulation results of the different $NO_3$–N processes for a 30-year weather data period are shown in Fig. 8. The scenario analysis of the long-term model indicates that the DRAINMOD model, after calibration and validation, can be a useful fertilizer-management tool in predicting for a long period the nitrate transport and transformation in the soil profile and the nitrate leaching to groundwater and surface waters. Furthermore, the model can be used as an environmental control when the environmental

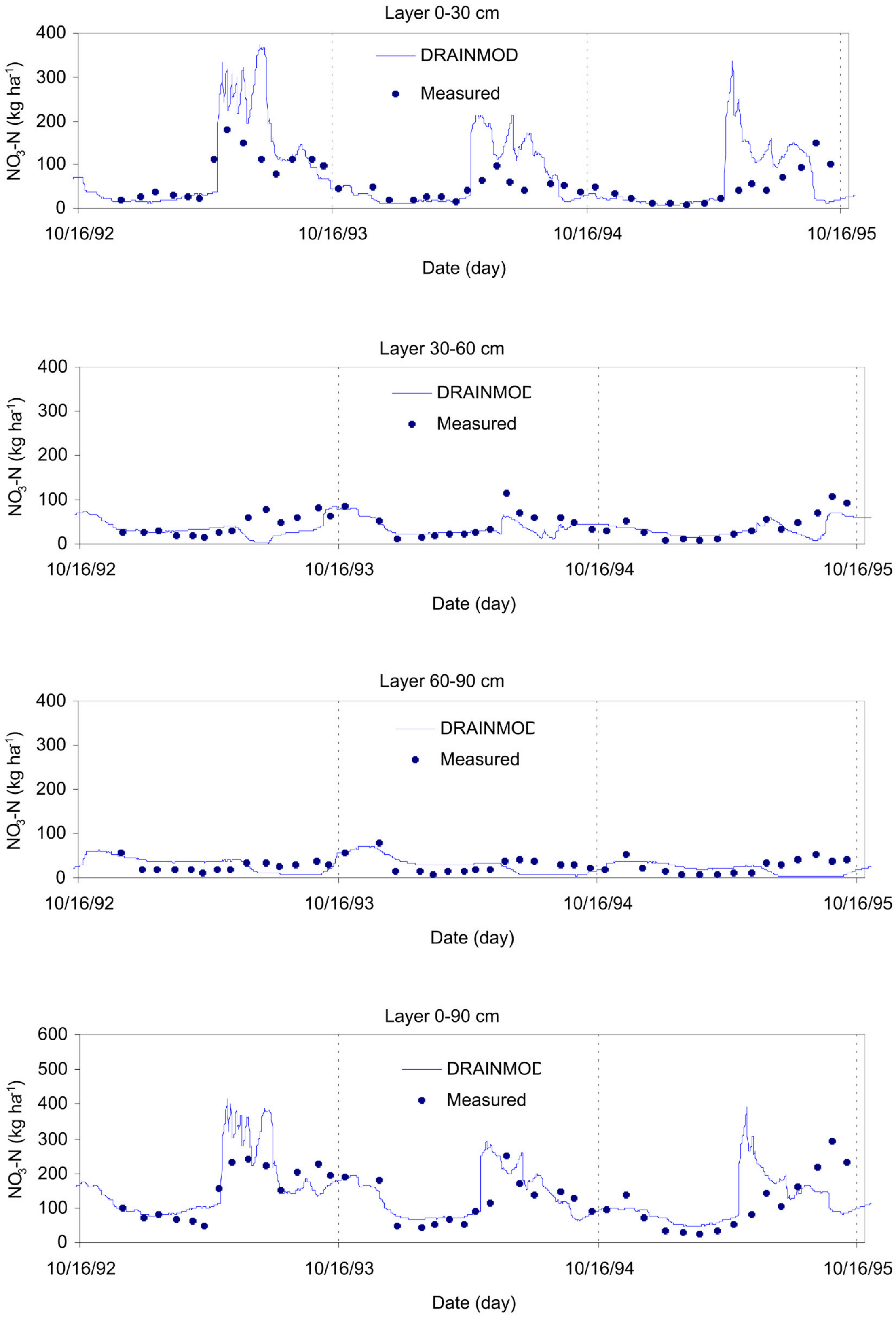

**FIGURE 5.** $NO_3$–N content in the soil profile at different depths for the maize field in Geel with a pig slurry application in the spring of 30 ton ha$^{-1}$ (scenario 3).

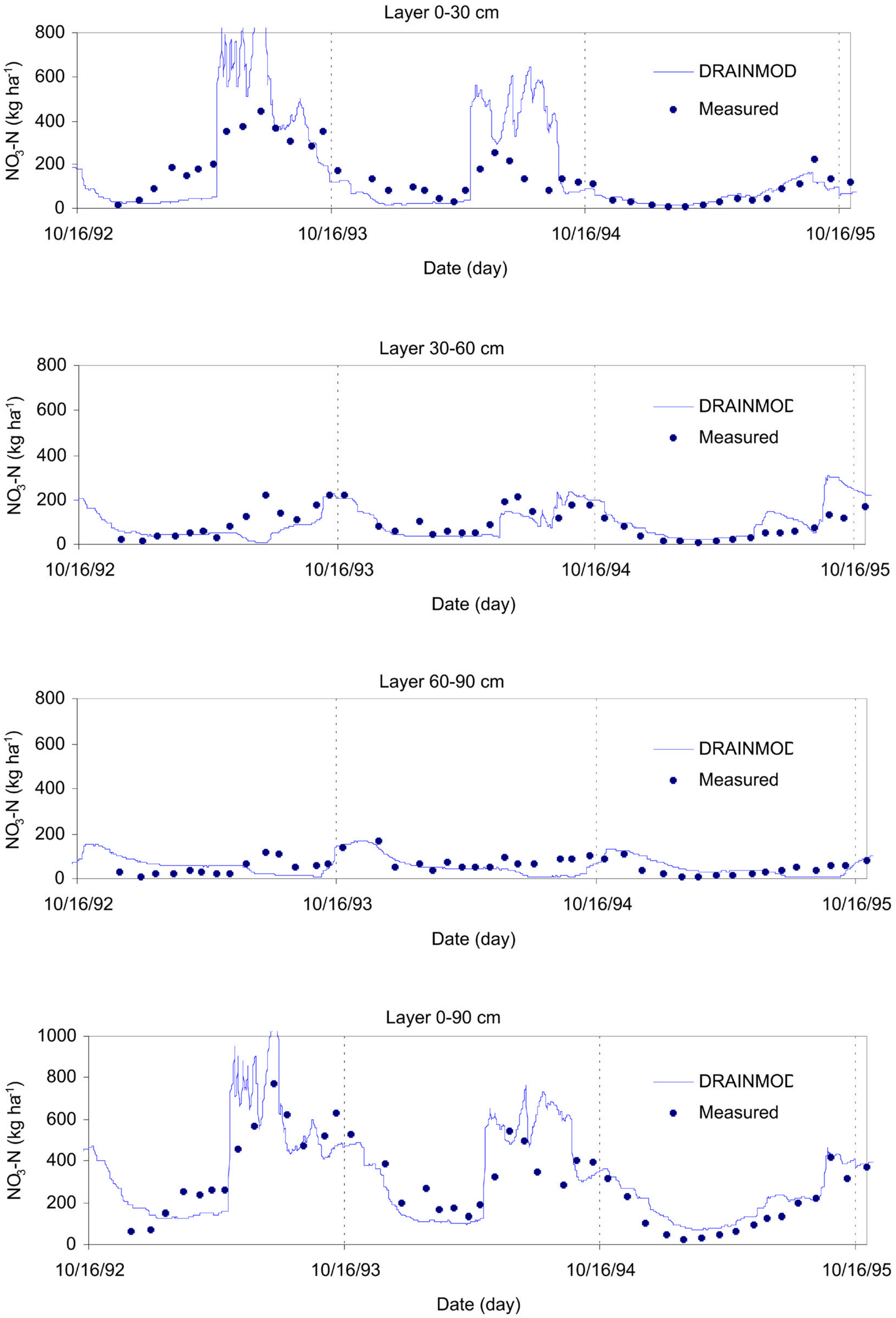

**FIGURE 6.** $NO_3$–N content in the soil profile at different depths for the maize field in Geel with a pig slurry application of 60 ton ha$^{-1}$ applied in the autumn and 60 ton ha$^{-1}$ applied in the spring (scenario 5).

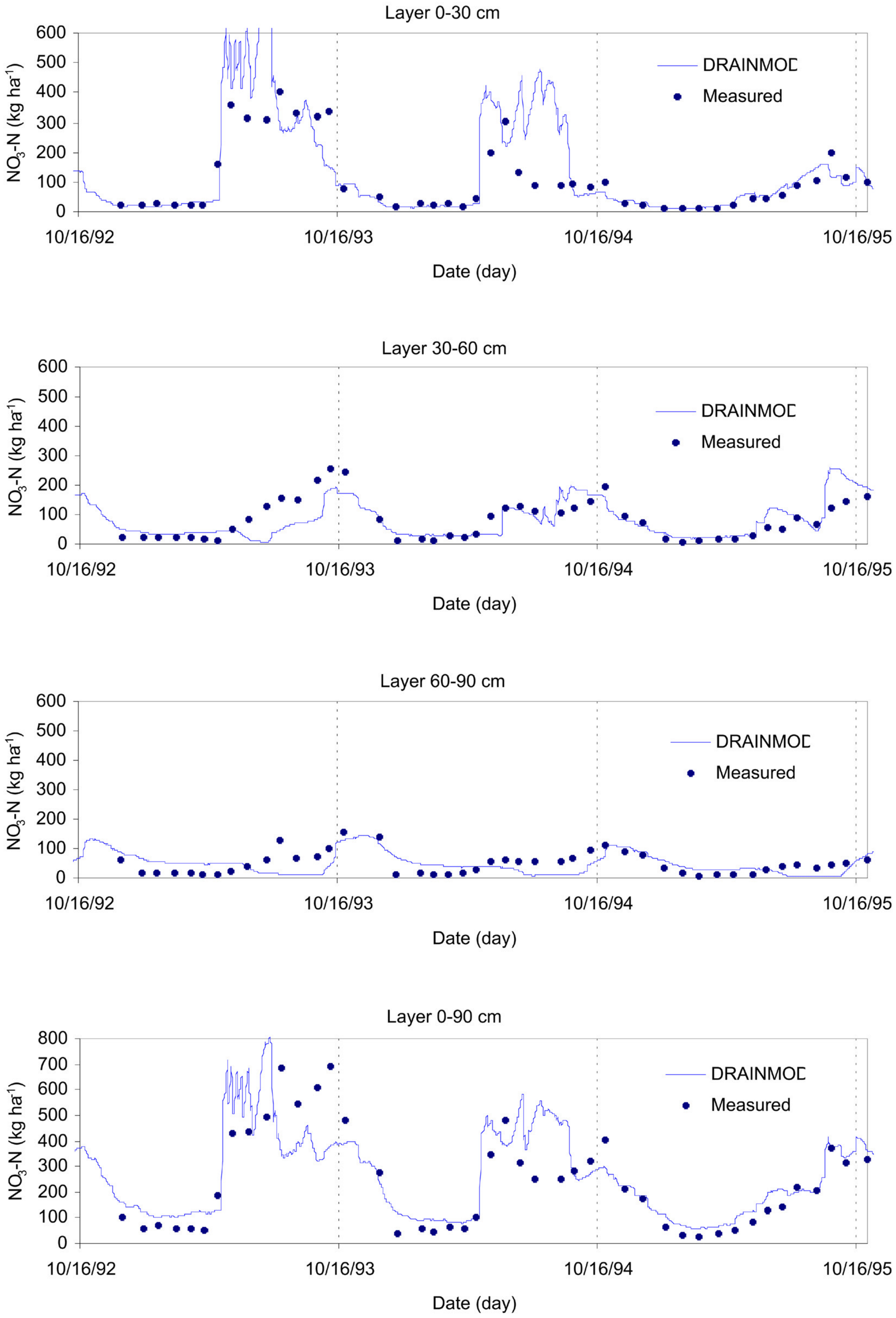

**FIGURE 7.** $NO_3$–N content in the soil profile at different depths for the maize field in Geel with a pig slurry application in the spring of 120 ton $ha^{-1}$ (scenario 4).

**TABLE 4**
**The Goodness of Fit ($R^2$) and Root Mean Square Residual (RMSR) at 30- to 60-cm Depth and Bottom of the Root Zone (90 to 120 cm)**

| | 30- to 60-cm depth | | Bottom of the Root Zone | |
|---|---|---|---|---|
| **Scenario** | **RMSR** | **$R^2$** | **RMSR** | **$R^2$** |
| Calibration: Scenario 3 | 0.0568 | 0.74 | 0.0728 | 0.84 |
| Validation: Scenario 5 | 0.0190 | 0.75 | 0.0221 | 0.85 |
| Scenario analyses | | | | |
| Scenario 1 | 0.0486 | 0.72 | 0.0383 | 0.80 |
| Scenario 2 | 0.0351 | 0.60 | 0.0228 | 0.85 |
| Scenario 4 | 0.0118 | 0.87 | 0.0137 | 0.92 |

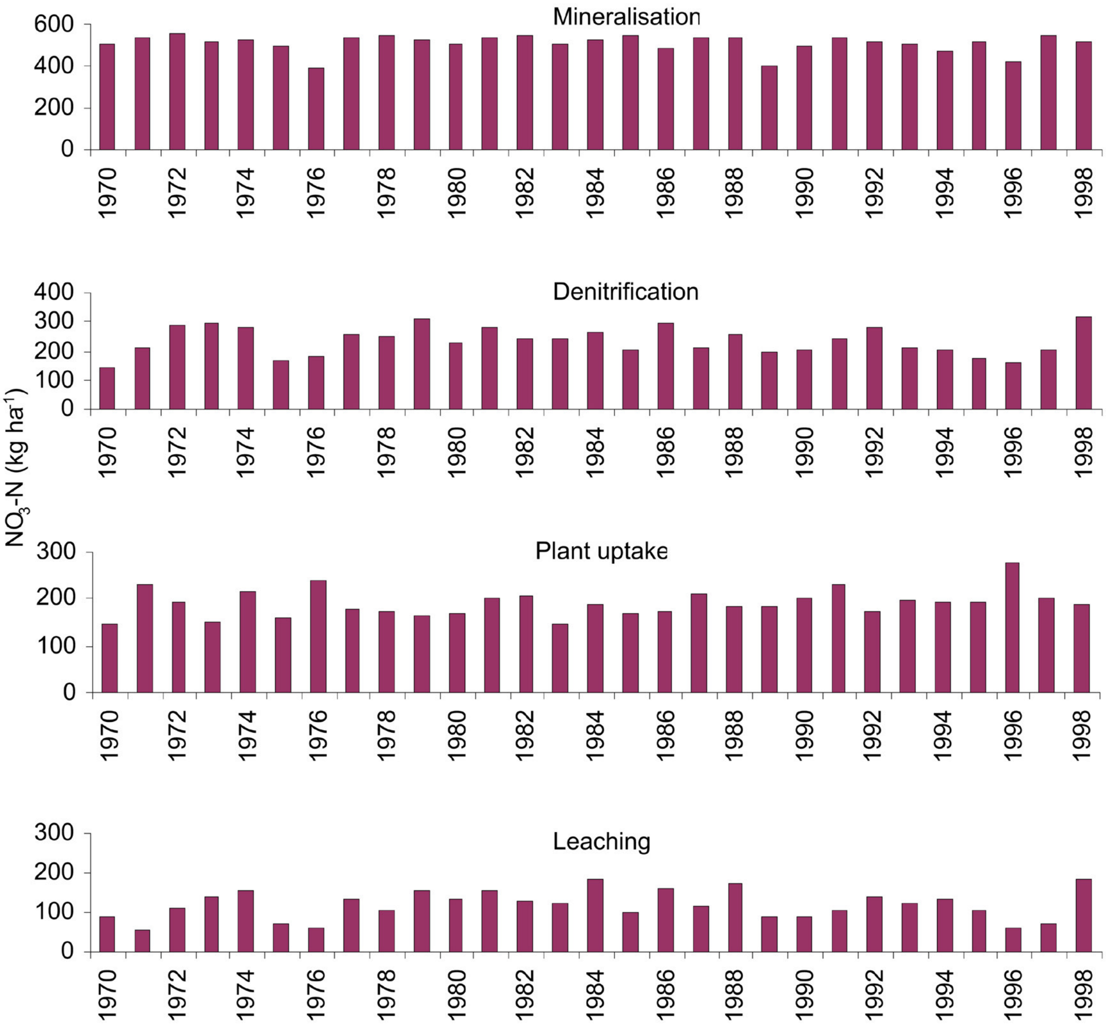

**FIGURE 8.** Scenario analysis results of different $NO_3$–N processes for a long simulation period (1969–1998).

objective has a greater importance than profits in agriculture. Finally, the method of analysis as presented here might be an interesting tool to examine quantitatively the nitrate leaching of soils rich in organic matter content.

## CONCLUSIONS

In western Europe, the current nitrogen use leads to considerable $NO_3$–N losses through leaching. In this study, the DRAINMOD model was used to predict the nitrogen transport and transformation within the soil profile and the $NO_3$–N leaching to ground- and surface waters. For the scenario analysis, climate data for a 30-year period, collected between 1969 and 1998 on an experimental field in Flanders, Belgium, was used. The DRAINMOD-N model requires limited input. To run the latter model one needs only time series of daily rainfall, evapotranspiration, maximum and minimum temperature, and few fertilizer application data together with site-specific parameters that are relatively easy to derive. Another important feature of the DRAINMOD-N model is that it simulates the lateral subsurface flow of water and $NO_3$–N to field drains, ditches, or drain tubes. The model can be used to help define (as a function of the local conditions, the crop cultivated, and the climate) the fertilizer package composed of organic and inorganic fertilizers that does not exceed the leaching limits imposed by environmental regulations. For economic viability of mixed farms in particular, it is important to know the maximum amounts of organic manure that can be applied safely on the different fields and to adjust to this application the use of chemical fertilizers. Furthermore, the model can be used as an environmental control when the environmental objective has a greater importance than the agricultural profits.

## ACKNOWLEDGMENTS

The authors would like to thank professor R.W. Skaggs from North Carolina State University for making available the PC version of the DRAINMOD model, including valuable suggestions for the correct use of the model and proper interpretation of modeling results.

| | | |
|---|---|---|
| Received: | July | 28, 2001 |
| Revised: | October | 29, 2001 |
| Accepted: | October | 30, 2001 |
| Published: | December | 1, 2001 |

## BIOSKETCH

**Alaa Abdallah Mohamed El-Sadek** is a Researcher at the Institute for Land and Water, Management, KULeuven, Belgium, and the National Water Research Center, Cairo, Egypt. He received a Ph.D. in Civil Engineering, K.U.Leuven, Belgium, an M.Sc. with Distinction in Water Resources Engineering, K.U.Leuven & V.U.B (Interuniversity Programme), Belgium (1998), an M.Sc. with Honours in Civil Engineering, Egypt (1996), a Postgraduate diploma with Honours in Civil Engineering, Egypt (1991), and a B.Sc. with Honours in Civil Engineering, Egypt (1989). Dr. El-Sadek was awarded the best young professional, National Water Research Center, Sharm El-Sheikh, Egypt in November 2000. His research focuses on irrigation and drainage, water quality control, nitrate transport in soil, nitrate leaching to surface and groundwater, river water quality, and solutes transport. He is also interested in nitrate modelling from the source on the soil surface, through the soil zone and the groundwater to the river, hydrological modelling of nitrate transport and transformation in combination with GIS, and scale problem in water resources management and hydrological modelling.
